# Application and advantages of induced sputum cytology with gradient concentrations of hypertonic saline in the diagnosis of chronic cough

**DOI:** 10.12669/pjms.42.3.13191

**Published:** 2026-03

**Authors:** Wei Luo, Hanxiao Xin, Pengyun Zhao, Ji Qi

**Affiliations:** 1 Wei Luo Blood Transfusion Department, Baoding No.1 Central Hospital, Baoding 071000, Hebei, China; 2 Hanxiao Xin Blood Transfusion Department, Baoding No.1 Central Hospital, Baoding 071000, Hebei, China; 3 Pengyun Zhao Blood Transfusion Department, Baoding No.1 Central Hospital, Baoding 071000, Hebei, China; 4 Ji Qi Department of Clinical Laboratory, Affiliated Hospital of Hebei University, Baoding 071000, Hebei, China

**Keywords:** Chronic Cough, Diagnosis, Gradient concentrations, Sputum Induction

## Abstract

**Objective:**

To explore the application and advantages of induced sputum cytology with gradient concentrations of hypertonic saline in the diagnosis of chronic cough (CC).

**Methodology:**

This was a prospective study. A total of 180 patients with CC admitted to Baoding No.1 Central Hospital between January 2022 to November 2024 were selected, and 115 individuals undergoing physical examinations during the same period were selected as the control group. Induced sputum cytology with gradient concentrations of hypertonic saline was conducted for all subjects. Analyzed the success rate of sputum induction and compared the results of sputum cytology examination of patients with different etiologies and the diagnosis rate of each test item for different etiologies.

**Results:**

The success rate of sputum induction was 92.78%(167/180), and the incidence of adverse reactions during the induction was 9.44%(17/180) in the patients with CC. These adverse reactions were resolved spontaneously after the induction was terminated. In patients with successful sputum induction, etiology was definitely diagnosed in 93.41%(156/176) patients, including cough variant asthma (CVA) in 38/156 patients (24.36%), eosinophilic bronchitis (EB) in 14/156 patients (8.97%), upper airway cough syndrome (UACS) in 25/156 patients (16.03%), gastroesophageal reflux cough (GERC) in 16/156 patients (10.26%), and atopic cough (AC) in 63/156 patients (40.38%). The etiology was unknown in 11/167 patients (6.59%).

**Conclusion:**

The success rate of sputum induction is high and the etiology is comprehensively diagnosed in patients with CC when induced sputum cytology with gradient concentrations of hypertonic saline is used.

## INTRODUCTION

Chronic cough (CC) is a respiratory disease commonly encountered in clinical practice, with complex etiologies and great difficulty in clinical diagnosis. Patients with CC often have poor prognosis due to insufficient symptomatic treatment.^1^-^3^ The application of tests, including bronchial provocation and peak expiratory flow tests, is relatively limited, and their diagnostic accuracy and sensitivity are generally not ideal. Therefore, it is extremely important to explore and find a method that can identify the different causes of CC to provide early symptomatic treatment and improve the prognosis of patients.

In recent years, induced sputum cytology has gradually been used to identify the etiologies of CC. Sufficient and qualified sputum can be induced and obtained in most patients with little or even no sputum by using hypertonic saline nebulization for cytology examination.^4^,^5^ To clarify the role of this examination in the diagnosis of CC, increasing concentrations of hypertonic saline were used to induce sputum in the present study. The success rate of sputum induction was observed and the differences in cytological classification of induced sputum in patients with CC of diverse etiologies were analyzed to provide references for the symptomatic treatment of these patients.

## METHODOLOGY

This was a prospective study. One hundred and eighty patients with CC admitted to Baoding No.1 Central Hospital from January 2022 to November 2024 were selected, and 115 individuals undergoing physical examinations during the same period were selected as the control group.

### Ethical Approval:

The study was approved by the Institutional Ethics Committee of Baoding No.1 Central Hospital (No.:2020055; Dated: May 25, 2020), and written informed consent was obtained from all participants.

### Inclusion criteria:

- With cough for at least eight weeks and being the only symptom at hospital visit.
- With no abnormalities found in chest X-ray or lung CT examination.
- With insufficient amount of sputum naturally.

### Exclusion criteria:

- With missing clinical data.
- With a history of respiratory infections within the past three months.
- With chronic respiratory diseases.
- Who were pregnant or lactating during the study.

### Cytology examination of sputum induced by hypertonic saline:

Induced sputum cytology with gradient concentrations of hypertonic saline was conducted. Forced vital capacity (FVC), forced expiratory volume in the first second (FEV1), FEV1/FVC, and peak expiratory flow (PEF) were measured before induction. After the mouth was repeatedly rinsed with sterilized water, 200μg salbutamol aerosol (manufacturer: Glaxo Wellcome, S.A., approval number: Imported drug registration number: H200900514, strength: 200 puffs per bottle, 0.14mg salbutamol per puff) was administered by oral inhalation. After inhalation of salbutamol aerosol, 3% hypertonic saline solution was nebulized using an air compression pump and inhaled for 10 minutes, and the patients were instructed to cough up sputum into a sterile culture dish when they felt it. Next, 4% hypertonic saline solution was nebulized and inhaled for 10 minutes, and the patients were asked to cough up sputum into a sterile culture dish. Finally, 5% hypertonic saline solution was nebulized and inhaled for 10 minutes, and the patients were asked to cough up sputum as much as possible into a sterile culture dish. The whole process of sputum induction lasted for 30 min. The sputum induction was immediately terminated if any of the following situations occurred:

- the amount of sputum in the sterile culture dish reached the amount required for the test, i.e., >200mg;
- the patients felt uncomfortable;
- 30 minutes of nebulization time was reached; and (4) the reduction of PEF was >20%.

### Sputum processing:

The sputum was weighed, and four volumes of 0.1% trypsin solution (manufacturer: Shanghai Rongbai Biological Technology Co., Ltd., item number: RS-40105, strength: 100ml) was added, mixed, and incubated in a water bath at 37°C for 10 mins. The mixture was vortexed twice for five seconds each time during the incubation to fully dissociate the sputum. The mixture was filtered with a 300-mesh filter to remove impurities, and cell suspension (about 100 μl) was then obtained. Total cell count and differential cell count were recorded using cell smears under an optical microscope.

### Cell analysis of sputum:

- *Total cell count:* 10μl cell suspension was taken with a pipette to prepare cell smears, and the total number of cells was counted using a 20x objective lens.
- *Cell viability:* 20μl of cell suspension was mixed with 20μl 0.4% trypan blue solution, the number of dead and live cells was counted within three min using a blood cell counter, and cell viability was calculated.
- *Differential cell count on cell smears:* The cell smears were fixed with neutral formaldehyde and stained with hematoxylin eosin (HE) solution. Twenty cells in a fixed field (400 ×) were randomly selected from each cell smear, a total of 400 inflammatory cells (eosinophils, neutrophils, lymphocytes, and macrophages) were counted, and the proportion of each cell type was calculated. The acceptance criterion of sputum included the proportion of squamous epithelial cells ≤10% and a viability of ≥80%. The adverse reactions during sputum induction were recorded.

### Statistical Analysis:

SPSS 19.0 software was used for data analysis. Enumeration data were presented as rate (%), and χ^2^ test was used for comparison between groups. The confidence interval was 95%. Measurement data (total cell count, and differential cell count) were not normally distributed, and presented as M (P_25_, P_75_). Mann-Whitney U test was used for the comparison between the two groups, and Kruskal-Wallis H test was used for comparison among multiple groups. Differences with a p<0.05 were considered statistically significant.

## RESULTS

One hundred and eighty patients with CC the BPT, total immunoglobulin E(IgE) test, and allergen test were conducted for all subjects. Among the 180 patients included, there were 86 males and 94 females, with an average age of 39.87±11.02 years old (range: 18 to 60 years). One hundred fifteen healthy individuals during the same period, including 53 males and 62 females, with an average age of 39.44±10.35 years old (range 18-60 years). There were no statistically significant differences in sex and age between the two groups (P>0.05).

Sputum induction was performed in 180 patients with CC, and sufficient and qualified sputum specimens were obtained from 167 out of 180 patients, with a success rate of sputum induction of 92.78% (167/180). Seventeen adverse reactions, including seven cases of pharyngeal itching, five nausea, four dizziness and one chest tightness, were reported in the process of sputum induction, and the incidence of adverse reactions was 9.44% (17/180). These adverse reactions were mild in severity and resolved spontaneously after the induction was terminated. In 167 patients with successful sputum induction, 156 patients (93.41%) were definitely diagnosed, including cough variant asthma (CVA) in 38 patients (24.36%), eosinophilic bronchitis (EB) in 14 patients (8.97%), upper airway cough syndrome (UACS) in 25 patients (16.03%), gastroesophageal reflux cough (GERC) in 16 patients (10.26%), and atopic cough (AC) in 63 patients (40.38%). The etiology was unknown in 11 patients (6.59%).

There were statistically significant differences in the total cell count and differential cell count in induced sputum among different groups (P<0.05). The total cell count was significantly increased in the sputum of patients with EB and AC(P<0.05), the proportion of eosinophils was significantly increased in the sputum of patients with CVA and EB(P<0.05), the proportion of neutrophils was significantly increased in the sputum of patients with EB, UACS, GERC and AC(P<0.05), the proportion of lymphocytes was significantly increased in the sputum of patients with GERC(P<0.05), and the proportion of macrophages was significantly decreased in the sputum of patients with CVA, EB, UACS, GERC and AC compared with those of the control group, respectively(P<0.05). The total cell count and the proportion of neutrophils were significantly increased and the proportion of eosinophils was significantly decreased in the sputum of patients with EB compared with those of CVA patients, respectively(P<0.05) (Table-I).

**Table-I:** Comparison of induced sputum cytology examination in patients with different etiologies [M (P25, P75)].

Groups	n	Total cell count (×106/g)	Proportion of eosinophils (%)	Proportion of Neutrophils(%)	Proportion of lymphocytes(%)	Proportion of macrophages(%)
CVA	38	2.70(0.88, 4.03)	0.13(0.05, 0.21)^*^	0.37(0.20, 0.51)	0.02(0.00, 0.10)	0.37(0.28, 0.49)^*^
EB	14	4.13(1.55, 5.68)^*#^	0.09(0.02, 0.27)^*#^	0.56(0.29, 0.68)^*#^	0.03(0.01, 0.09)	0.33(0.22, 0.51)^*^
UACS	25	2.52(0.91, 3.64)	0.02(0.01, 0.09)	0.45(0.23, 0.57)^*^	0.04(0.01, 0.08)	0.42(0.30, 0.62)^*^
GERC	16	2.67(1.02, 4.71)	0.01(0.00, 0.23)	0.58(0.33, 0.76)^*^	0.05(0.02, 0.11)^*^	0.39(0.24, 0.57)^*^
AC	63	4.49(1.60, 5.14)^*^	0.02(0.00, 0.15)	0.50(0.31, 0.72)^*^	0.03(0.00, 0.07)	0.45(0.29, 0.66)^*^
Control	115	2.14(0.79, 3.87)	0.01(0.00, 0.17)	0.34(0.19, 0.44)	0.02(0.00, 0.08)	0.68(0.35, 0.81)
H		70.596	63.824	25.458	37.991	42.036
P		0.000	0.000	0.000	0.000	0.000

***Notes:***

* P<0.05, versus the control group;

# P<0.05, versus the CVA group.

A proportion of eosinophils ≥3% in sputum was of high value in the diagnosis of EB and some value in the diagnosis of CVA. The positive BPT, however, was a prerequisite for the diagnosis of CVA. The diagnostic criteria of AC included a low proportion of eosinophils in sputum, negative BPT, positive allergen test, and a history of urticaria and allergic rhinitis. Almost all UACS patients had rhinitis and sinusitis secretions flowing back to the throat to stimulate a dry cough. Increased serum total IgE was more common in CVA and AC (Table-II).

**Table-II:** Comparison of the diagnosis rate of each test item in patients with different etiologies[n(%)].

Groups	n	Proportion of eosinophils ≥3%	BPT	Total IgE test	Allergen test
CVA	38	18(47.37)	32(84.21)	31(81.58)	25(65.79)
EB	14	8(57.14)	0(0.00)	0(0.00)	0(0.00)
UACS	25	5(20.00)	0(0.00)	7(28.00)	8(32.00)
GERC	16	3(18.75)	0(0.00)	1(6.25)	0(0.00)
AC	63	4(6.35)	0(0.00)	39(63.93)	62(98.41)

## DISCUSSION

In the present study, the success rate of sputum induction in patients with CC was 92.78%, with a favorable safety profile. Few adverse reactions with mild symptoms were reported during the induction process, and resolved spontaneously after the induction was terminated. Induced sputum cytology can objectively reflect the airway inflammation status in patients. Interest in this test is increasing because of its advantages of simple operation and non-invasiveness.^6^ In the process of sputum induction with gradient concentrations of hypertonic saline nebulization, increasing concentrations of hypertonic saline (3% - 4% - 5%) were inhaled by the subjects in a fixed time period, and the concentration of hypertonic saline was gradually increased dependent on the conditions of cough and lung ventilation in the subjects to allow them to cough up sufficient amount of sputum for examination and consequently improve the success rate of sputum induction.^7^ CVA is a special type of asthma with CC as the primary or only symptom, and is characterized by persistent airway inflammatory response and a certain degree of airway hyperresponsiveness.^8^,^9^ Induced sputum cytology directly reflects the presence and changes of eosinophilic inflammation in patients with CVA, playing an important role in airway assessment.^10^ Gao J et al.^11^ found that the proportion of eosinophils in the induced sputum of patients with CVA was closely related to the severity of airway hyperresponsiveness. The increased proportion of eosinophils in sputum may seriously damage the bronchial tube wall, increase airflow obstruction, and significantly aggravate the severity of cough in patients.

The results of the present study showed that the proportion of eosinophils was significantly increased, and the proportion of macrophages was significantly decreased in the sputum of patients with CVA compared with those in the control group, which was similar to the findings of Gao J et al.^11^, suggesting that the increased proportion of eosinophils and the decreased proportion of macrophages in sputum were probably related to the development of CVA. EB is a non-asthmatic bronchitis characterized by an elevated eosinophil count in the sputum without airway hyperresponsiveness and airflow obstruction, and it is considered one of the causes of CC.^12^,^13^ It was found in the present study that in patients with EB, the total cell count and the proportions of eosinophils and neutrophils were significantly increased and the proportion of macrophages was significantly decreased in sputum compared with those of the control group, and the total cell count and the proportion of neutrophils were significantly increased and the proportion of eosinophils was significantly decreased compared with those in patients with CVA, suggesting that the induced sputum cytology with gradient concentrations of hypertonic saline can be used as an effective method for the differential diagnosis of CVA and EB. UACS refers to cough caused by nasal diseases. Allergic inflammation in the nasal mucosa can directly stimulate the cough receptors in the upper respiratory tract to induce airway hyperresponsiveness and pathological cough.^14^-^16^ GERC is a clinical syndrome characterized by cough, and some patients with GERC do not present with typical reflux symptoms, making it difficult to diagnose.^17^,^18^ The results of the present study showed that the proportion of neutrophils in the sputum of patients with UACS and GERC was significantly increased and the proportion of macrophages was significantly decreased compared with those of the control group, suggesting that UACS and GERC are airway inflammatory diseases dominated by neutrophils and macrophages.

At the same time, the proportion of lymphocytes in the sputum of patients with GERC was much higher than that of the control group, which may serve as the basis for the differential diagnosis of UACS and GERC. AC, with the main clinical manifestation of dry cough, is an atopic disease. Similar to EB, AC is not associated with airway hyperresponsiveness and reversible airflow obstruction, and the differential diagnosis of AC and EB mainly relies on the allergen test.^19^ The results of the present study demonstrated that the total cell count and the proportion of neutrophils in the sputum of patients with AC were significantly higher and the proportion of macrophages was significantly lower than those of the control group, and the differences versus EB were not significant. This was possibly related to the small sample size in the present study, and further studies are needed. Studies^20^ have found that the level of serum total IgE significantly decreased in children with CVA after being treated with montelukast sodium combined with budesonide, and serum total IgE may serve as one of the bases for the diagnosis of CVA. In the present study, Analysis of percentage of eosinophils, BPT, blood total IgE test, and allergen test showed that the proportion of eosinophils ≥3% in sputum was of high value in the diagnosis of EB and some value in the diagnosis of CVA. The positive BPT, however, was a prerequisite for the diagnosis of CVA. The diagnostic criteria of AC included a low proportion of eosinophils in sputum, negative BPT, positive allergen test, and a history of urticaria and allergic rhinitis.

### Limitations:

However, this study also has some shortcomings, such as a small number of samples. In view of this, more samples should be included in future studies to further validate the findings of this study.

## CONCLUSIONS

Induced sputum cytology findings can be used as a basis for the diagnosis of CC with different etiologies, and a combination of these findings with BPT, blood total IgE test and allergen test may be more beneficial to a better diagnosis of etiologies in patients with CC in clinical practice.

### Authors’ Contributions:

**WL** and **JQ:** Carried out the studies, data collection, drafted the manuscript, and are responsible and accountable for the accuracy or integrity of the work.

**HX** and **PZ:** Study design, statistical analysis

All authors have read and approved the final manuscript.
